# Postnatal epigenetic differences in calves following transient fetal infection with bovine viral diarrhea virus

**DOI:** 10.1186/s12864-025-11562-5

**Published:** 2025-05-02

**Authors:** Jessica N. Kincade, Terry E. Engle, Marcela Henao-Tamayo, Jordan M. Eder, Erin M. McDonald, Darcy M. Deines, Brie M. Wright, Dilyara Murtazina, Jeanette V. Bishop, Thomas R. Hansen, Hana Van Campen

**Affiliations:** 1 Department of Biomedical Sciences, Colorado State University, Fort Collins, CO USA; 2 Department of Animal Sciences, Colorado State University, Fort Collins, CO USA; 3 Department of Microbiology, Immunology, and Pathology, Colorado State University, Fort Collins, CO USA; 4 Research Innovation Center, Fort Collins, CO USA

**Keywords:** BVDV, Epigenetics, Methylation, Transient infection, Fetal infection

## Abstract

**Background:**

Bovine viral diarrhea virus (BVDV) is the most detrimental pestivirus within the cattle industry. Infection with vertically transmissible BVDV prior to 125 days of gestation results in the generation of a persistently infected (PI) calf. These PI calves are unable to clear the virus in utero, due to an incomplete immune response. However, when infection with BVDV occurs after 150 days of gestation, the fetus clears the transient infection (TI) in utero and is born with antibodies specific to the infecting strain of BVDV. Variations in DNA methylation have been identified in white blood cells (WBC) from TI heifers at birth. It was hypothesized that epigenomic alterations persist into the postnatal period and contribute to previously undocumented pathologies. To study these possible effects, DNA was isolated from the WBCs of 5 TI heifers and 5 control heifers at 4 months of age and subjected to reduced representation bisulfite sequencing (RRBS).

**Results:**

Differential analysis of the methylome revealed a total of 3,047 differentially methylated CpG sites (DMSs), 1,349 of which were hypermethylated and the other 1,698 were hypomethylated. Genes containing differential methylation were associated with inflammation, reactive oxygen species (ROS) production, and metabolism. Complete blood count (CBC) data identified a higher lymphocyte percentage in TI heifers. When compared in the context of the CD45^+^ parent population, spectral flow cytometry revealed increased intermediate monocytes, B cells, and CD25^+^/CD127^−^ T cells, and decreased CD4^+^/CD8b^+^ T cells. Comparative analysis revealed differential methylation of CpG sites contained in 205 genes, 5 promoters, and 10 CpG islands at birth that were also present at 4 months of age. Comparison of differential methylation in TI heifers and PI heifers at 4 months of age showed 465 genes, 18 promoters, and 34 CpG islands in common.

**Conclusion:**

Differential methylation of WBC DNA persists to 4 months of age in TI heifers and is associated with dysregulation of inflammation, metabolism, and growth. Analysis of differential methylation in TI heifers contributes to the understanding of how fetal infection with BVDV induces postnatal detriments related to profit loss.

**Supplementary Information:**

The online version contains supplementary material available at 10.1186/s12864-025-11562-5.

## Introduction

Bovine viral diarrhea virus (BVDV) is a globally prevalent virus that causes billions of dollars in loss to the cattle industry. It is estimated that an outbreak of BVDV costs a cattle producer anywhere from $20 to $103 per infected animal [1, 2]. Acute infections resulting from horizontal transmission not only decrease the average daily weight gain and milk production but cause reproductive losses as well [1, 3–8]. Additionally, BVDV is one of the few viruses capable of crossing the placenta and infecting the fetus during gestation. The outcome of a fetal calf infected in utero depends largely on the developmental progress of the adaptive immune response [3, 9, 10]. If the infection occurs early in gestation, after 30 days of gestation but prior to 125 days of gestation, the fetus mounts an incomplete immune response and is unable to clear the virus [9, 11–15]. These calves, termed persistently infected (PI), are immunotolerant and serve as the primary reservoir for the virus due to their chronic shedding of the virus.

If fetal infection occurs after 150 days of gestation, the fetus mounts a more robust immune response. The generated immune response induces both innate and adaptive immunity, as evidenced by transcriptional activation of genes associated with inflammation and viral immunity (*DDX58, NFκB, IRF7, IFNB,* and *ISG15*) as well as those associated with T and B cells (*PSMB9, IFI30, CD4, CD8A, CD8B,* and *CD79B*) [11]*.* These calves, termed transiently infected (TI), produce antibodies specific to the infecting strain of BVDV and clear the virus prior to birth. Currently, there is no way to distinguish calves with BVDV TI in utero from those that experience TI with BVDV in the early postnatal period.

Environmental influences such as nutrition, stress, and disease can affect the postnatal health and development of an organism, per the developmental origin of health and disease (DOHaD) theory. One study of postnatal effects following fetal TI found calves to be 2.3 times more likely to develop an illness requiring treatment [16], while another demonstrated lower body weight in suspected TI calves [17]. It has recently been shown by our laboratory that during a feed trial, heifers infected with BVDV during late fetal development (TIs) have a lower entry and exit body weight as well as decreased average daily gain [18]. During the feedlot trial, TI heifers also exhibited signs of increased inflammation which include decreased glutathione, increased oxidized glutathione, and increased plasma ceruloplasmin concentrations [18]. We have previously demonstrated that TI heifers have lower mean body weight and differential methylation of white blood cell (WBC) DNA in genes associated with the immune system, growth, and development at birth [19]. However, little remains known about the postnatal epigenetics of fetal TI calves.

Methylation of DNA in mammals occurs most often on cytosines that are immediately followed by guanines [20]. These cytosine-phosphate-guanine (CpG) motifs are known to enhance transcriptional binding when not methylated, but in striking contrast, methylation of these sites is known to effectively silence gene transcription [21]. Other viruses within *Flaviviridae* such as hepatitis c virus and Zika virus are known to evade immune detection through manipulation of epigenetic mechanisms [22, 23]. Differential methylation of splenic tissue and WBCs has been demonstrated in PI calves at day 245 of gestation and 4 months of age, respectively [24, 25]. Moreover, differential methylation has been identified in TI calves at birth [19]. Because gestation is a time of high fetal epigenetic plasticity, it is possible that normal fetal epigenetic programming is disrupted by fetal BVDV infection. Per the DOHaD theory, it was then hypothesized that epigenetic alterations in TI fetal DNA due to fetal BVDV infection persist into the postnatal period and continue to impact immune function and growth.

In this study, fetal TI and control heifers were generated by inoculating seronegative pregnant heifers on day 175 of gestation with a non-cytopathic (ncp) strain of BVDV type 2 or sham-inoculated with phosphate buffered saline. The methylation of WBC DNA in four-month-old heifers born from dams infected with BVDV during pregnancy was compared to that of uninfected controls. Complete blood counts (CBCs), flow cytometry analysis, and body weight demonstrate the state of the immune system and growth.

## Materials and methods

### Animals

Twenty-eight yearling, Hereford heifers were purchased from a private Montana ranch that does not vaccinate for BVDV. These heifers were housed at the Animal Research, Development, and Education Center (ARDEC) at Colorado State University (CSU) where they were vaccinated with Ultrabac 7 (Zoetis, Kalamazoo, MI, USA) upon arrival and one month later. Heifers were then moved to the Animal Reproduction and Biotechnology Laboratory (ARBL) at CSU for breeding. Estrous cycles were synchronized in yearling heifers using 14-day intravaginal progesterone devices (EAZI-BREED, Zoetis) and an intramuscular injection of prostaglandin F2α (Lutalyse HighCon, Zoetis). Estrus was detected using heat-detection patches (Estrotect, Spring Valley, WI, USA) and visual observation. Upon detection of estrus, heifers were artificially inseminated with X-bearing sperm from a single Angus bull (Select Sires MidAmerica, Logan, UT, USA). The pregnant dams were inoculated with BVDV or sham inoculated with phosphate buffered saline on day 175 of gestation. Pregnant heifers were vaccinated with ScourGuard 4 KC (Zoetis) at 6 and 3 weeks prior to the expected calving date. The dams of the calves generated were sold at auction after weaning. Control and TI heifer calves were maintained on a standard finishing ration at ARDEC until 4 months of age. Upon completion of the longitudinal study, heifers were euthanized humanely at 17 months of age using penetrative captive bolt followed by exsanguination at the JBS Global Food Innovation Center (GFIC) at CSU.

### Study design

To study the postnatal impact of fetal TI with BVDV, 12 of the heifers seronegative for BVDV were randomly selected for inoculation with 4.0 log_10_TCID_50_/mL of non-cytopathic BVDV 2 type 96B2222 at day 175 of pregnancy [19]. The other twelve dams were sham inoculated with phosphate buffered saline. Twelve female control pregnancies were carried to term. Twelve TI pregnancies were carried to term and resulted in the generation of our TI group; a single TI bull calf was excluded from the study to prevent skew of the data due to potential sex differences (n = 11). Heifers were assigned a number ID based on their birth date for the duration of the study. Blood samples from all heifers were analyzed for complete blood count and flow cytometry. At 4 months of age, WBCs were isolated from whole blood samples. The DNA was extracted from WBCs and utilized for reduced representation bisulfite sequencing (RRBS). A total of 10 samples were sent to Zymo Research for sequencing; 5 samples were selected at random from both the control and TI group.

### Hematology

Whole blood samples were collected in K_2_EDTA tubes (Becton, Dickinson and Company, Franklin Lakes, New Jersey, USA cat# 07417) for DNA, RNA, flow cytometry, CBCs (Heska Elemental 5), and immunoglobulin concentration testing. Samples were stored on ice until processing. Upon arrival at the ARBL, the whole blood samples were centrifuged at 1500 × g for 15 min at 4 °C to separate the serum, buffy coat, and red blood cells. The buffy coat was aspirated and transferred to a sterile 15 mL tube containing 5 mL of ammonium-chloride-potassium lysing buffer (ACK; KD Medical, Columbia, MD, USA, cat # TGF- 3015). Following a 5-min incubation at room temperature, centrifugation at 300 × g for 10 min at 4 °C was used to pellet the WBCs. The supernatant was discarded, and the pellet was resuspended in 2.5 mL of ACK lysing buffer. After another 5-min incubation at room temperature, the pelleting of the WBCs via centrifugation, and decanting of the supernatant, the pellet was resuspended in phosphate buffered saline at a pH of 7.4. The sample was pelleted again by centrifugation at 300 × g for 10 min at 4 °C, the supernatant was discarded, and the WBCs were resuspended in 1 mL of phosphate buffered saline. The samples were snap-frozen using dry ice and stored until DNA extraction. These methods are previously described in [19].

### Reduced representation bisulfite sequencing

White blood cell samples for RRBS were processed according to manufacturer’s instructions using the Qiagen DNeasy Blood and Tissue Kit (Qiagen, Germantown, Maryland, USA). Samples were shipped on dry ice to Zymo Research (Irvine, CA, USA) for paired end RRBS. Samples were digested using MspI (NEB; Ipswich, MA, USA) and purified with DNA Clean & Concentrator- 5 (Zymo Research). Per Illumina’s specifications, fragments were ligated to pre-annealed adapters with cytosine replaced with 5’-methyl-cytosine. Ligated fragments larger than 50 base pairs were recovered with DNA Clean & Concentrator- 5 (Zymo Research) and bisulfite treated with EZ DNA Methylation-Lightning Kit (Zymo Research). Using Illumina indices, samples were subjected to PCR and purified using DNA Clean & Concentrator- 5 (Zymo Research). Sample size and concentration were confirmed using the Agilent 2200 TapeStation and libraries were sequenced using a NovaSeq6000.

Zymo Research trimmed and filtered sequences shorter than 20 base pairs using TrimGalore (v0.6.4_dev). FastQC (v0.11.9) was used to determine quality Phred scores, per sequence GC content, and remove overrepresented sequences. Adapter contamination was below 0.1% in all samples. Samples were aligned to a bisulfite converted version of the most recent bovine reference genome from the University of California, Santa Cruz (UCSC; ARS-UCD1.2/bosTau9) using Bismark (v0.22.3). The average observed vs expected methylation level and the associated Pearson correlation coefficients were found using MethylDackel (v0.5.0). Additional details pertaining to quality control performed by Zymo Research can be found using GitHub username Zymo-Research within the ‘service-pipeline-documentation’ repository, folder ‘docs’, file ‘how_to_read_methylseq_report.basic.’

The average CpG coverage was 8X, the average quantity of unique CpGs per sample was 7.9 million. The average total number of reads was approximately 40 million per sample. The percent bisulfite conversion rate was greater than 99% for both non-CpG and spike-in reads. All mean quality scores (Phred) were greater than 30 at all base pairs. An average of 33% of reads mapped uniquely to a single sequence within the genome, an average of 51% mapped ambiguously to more than a single sequence within the genome, and an average of 16% did not align at all. Pearson correlation coefficients for observed vs expected methylation level were greater than 0.95 for all samples.

### Methylation bioinformatics and pathway analysis

Files received by Zymo Research were processed as raw.bam files using the methylKit package in R [26]. These files were aligned to the most recent bovine genome available through the UCSC (ARS-UCD1.2/bosTau9). Data was initially filtered to remove sites containing less than 10 reads or reads greater than the 99.9^th^ percentile. Data was normalized by median coverage, filtered by a standard deviation of 2, and assessed for outliers. To eliminate SNPs and potential genetic variance, only cytosines identified in all samples in both the treatment and control group were stored for analysis, as per methylKit. Logistic regression was used to identify singular CpG sites containing hypermethylation (positive meth.diff values) or hypomethylation (negative meth.diff values). Singular CpG sites were classified as differentially methylated if the site varied in methylation in at least 25% of reads in the treatment group (absolute value of meth.diff > 25) and the false discovery rate (FDR/q-value) was less than 0.01. Identified CpG sites containing differential methylation were annotated and mapped to genes using the most recent reference genome available from the UCSC (ARS-UCD1.2/bosTau9).

Subsequent analyses identified regions of the genome mapped either as promoters or CpG islands. Experimentally validated promoters included in the UCSC reference genome were mapped and analyzed for differential methylation. The UCSC also provides a publicly available reference track mapping areas likely to be CpG islands. These areas must have a length greater than 200 base pairs, a guanine-cytosine content of greater than 50%, and an observed-to-expected CpG ratio greater than 0.6. The methylKit package contains functions that summarize the methylation data contained within a specified region. The quantified summary of methylation within a specific region can then be compared between treatment groups to reveal differential methylation in regions with greater regulatory function. Logistic regression was utilized to compare the levels of methylation between control and treatment groups. The CpG islands and promoters were deemed differentially methylated if the absolute value of meth.diff > 15 and FDR/q-value < 0.01. Genes associated with differential methylation were annotated using commands from ‘genomation’, ‘clusterProfiler’, and ‘org.Bt.eg.db’ in R [27, 28]. Bioinformatic code is available online at GitHub user profile jnkincade repository Bovine-RRBS-Analysis.

Data resulting from differential analysis was examined using Ingenuity Pathway Analysis (IPA; Qiagen, Germantown, Maryland, USA). The IPA software contains pathways consisting of a list of genes pertaining to a specific function, disease, or process. The IPA software utilizes a fisher’s exact test to assign a p-value to each pathway based on the probability that the observed overlap between genes in the dataset and predetermined pathways are due to random chance. The IPA software also calculates a predictive value, a z-score, to each pathway based on the input data [29]. The canonical pathways were then sorted according to the absolute value of the z-score, from largest to smallest, with positive z-scores indicating activation and negative z-scores indicating inhibition. The machine learning (ML) disease pathways do not have an associated z-score and were instead sorted according to the largest -log(p-value). Predicted upstream regulators are identified in IPA based on how many known downstream targets are identified within the dataset. A prediction of activation or inhibition is made according to the observed differential measures of the identified downstream activators.

Previously analyzed data utilized for comparisons herein can be found within publications titled “Epigenetic Modifications of White Blood Cell DNA Caused by Transient Fetal Infection with Bovine Viral Diarrhea Virus” (DOI: 10.3390/v1605721) and “Epigenetic Alterations in Calves Persistently Infected with Bovine Viral Diarrhea Virus.” Details pertaining to the analysis of previously published datasets can be found within their respective publications but were identical to methods published herein. A file listing individual DMSs, along with promoter and CpG island differentially methylated regions (DMRs), was generated for the TI calves at 4 months of age and previously published datasets. The file includes the chromosome, start position, end position, gene symbol, gene name, REFSEQ, and ENTREZID in which the identified DMS was contained. The associated p-value, q-value, and meth.diff value were also listed. These files were merged according to gene symbol in R, allowing for comparison of DMSs within the same gene body. A secondary comparison was made to yield only DMSs with similar methylation status at both timepoints (*MIF* contained hypermethylated DMSs at birth in TI heifers and at 4 months). Genes containing multiple DMSs with opposing methylation (hyper and hypomethylation of DMSs within a single gene) were excluded from this comparison.

### Flow cytometry

Whole blood samples were collected in K_2_EDTA tubes and processed by Zoetis Inc.

Red blood cells were lysed using 1X RBC lysis buffer (eBiosciences, San Diego, CA, USA cat # 00–4300 - 54). After centrifugation at 500 × g for 5 min, the supernatant was discarded and the pellet was resuspended in 1 mL of Dulbecco’s phosphate buffered saline (DPBS; ThermoFisher, Waltham, MA, USA cat # 14,190–136). After adding an additional 10 mL of DPBS to the solution, the samples were centrifuged at 500 × g for 5 min. The supernatant was discarded, and the pellet resuspended in 1 mL of DPBS before filtering through a cell-strainer cap (Corning, Corning, NY, USA cat # 352,235). After a 1:20 dilution using DPBS, the concentration of viable cells was determined using a Vi-CELL (Beckman Coulter, Loveland, CO, USA). Viable cells were seeded into a 96 well plate at a concentration of 1 × 10^6^ cells per well. Remaining cells were frozen in a solution of Serum Free Cell Freezing Medium (ATCC, Manassas, VA, USA cat # 30–2600) and stored at −80 °C. Each well containing cells in the 96 well plate was brought to a final volume of approximately 200–300 µL with DPBS. The plate was centrifuged, supernatant discarded, and the cell pellets resuspended in 100 µL of Fixable Viability Stain 620 (BD Biosciences, Franklin Lakes, NJ, USA cat # 564,996) at a 1:1000 concentration. After a dark, 10-min incubation on ice, 200 µL of fluorescence-activated cell sorting (FACS) buffer (BD Biosciences, cat # 554,657) was added to each well. The plate was centrifuged, the supernatant discarded, and the cells were resuspended in 100 µL of the staining master mix. The cells were incubated in the staining solution for 20 min in the dark, on ice. Another 200 µL of FACS buffer was added before the plate was centrifuged and the supernatant was discarded. Cells were washed using 300 µL of FACS buffer, centrifuged, and the supernatant was discarded. Cells were resuspended in 100 µL of diluted stabilizing fixative (1:3; BD Biosciences, cat # 338,036) and incubated at room temperature for 5 min. Lastly, 100 µL of FACS buffer was added to each well, the plate was centrifuged, the supernatant was discarded, and the cells were resuspended in 200 µL of FACS buffer. Samples were held in the dark at 4 °C until analysis. Cell samples were run at the CSU Flow Cytometry Core using a 4L 16 V- 14B- 10YG- 8R Cytek Aurora spectral cytometer.

Data was gated using FlowJo (BD Biosciences, Franklin Lakes, NJ, USA). Flow cytometry data in the form of frequency relative to the parent population of either CD45 positive cells or CD3 positive cells was compared in GraphPad Prism 10 (GraphPad Software, San Diego, CA, USA).

### Statistical analysis

Statistical analyses were performed in GraphPad Prism 10 (GraphPad Software, San Diego, California, USA). Values within the control or treatment group were first tested for normality. Outliers were identified and removed prior to analysis but are denoted in figures as a hollow circle. Data with normal distribution was analyzed using an unpaired parametric t-test. Because the control group contained values from 12 heifers and the treatment group contained values from 11 heifers, Welch’s correction was applied. Data that was not normally distributed was subjected to a non-parametric Mann–Whitney test. Differences between controls and TI heifers were considered significant if p < 0.05. Data are presented as mean ± standard error of the mean (SEM).

## Results

### Generation of TI calves and body weight

**Fig. 1:**
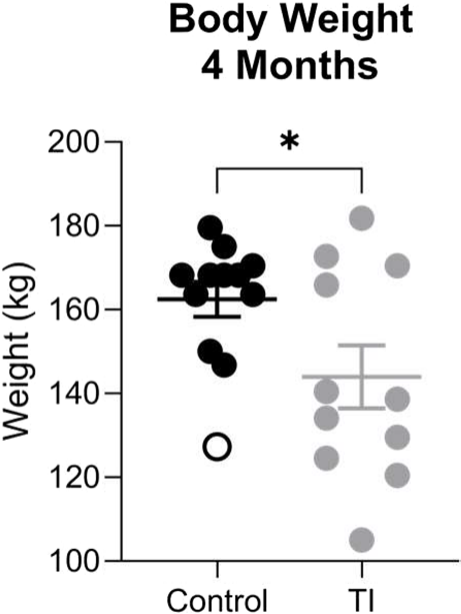
Body weight is decreased in TI heifers at 4 months of age. Quantification of body weight in kilograms of control and TI heifers at 4 months of age. A single outlier (Control—127.27 kg) was excluded from significance testing but is denoted within the figure as a hollow circle. Following removal of the outlier, the data passed a Shapiro–Wilk normality test. Data are displayed as mean ± standard error of the mean (SEM). Asterisks denote the significance between groups, ** *P* < 0.01

At 4 months of age, the average body weight of the TI heifers was lower than the average body weight of the control heifers (165.6 ± 2.918 kg vs 144 ± 7.53 kg; p < 0.01; Fig. 1).

### DNA Methylation

**Fig. 2:**
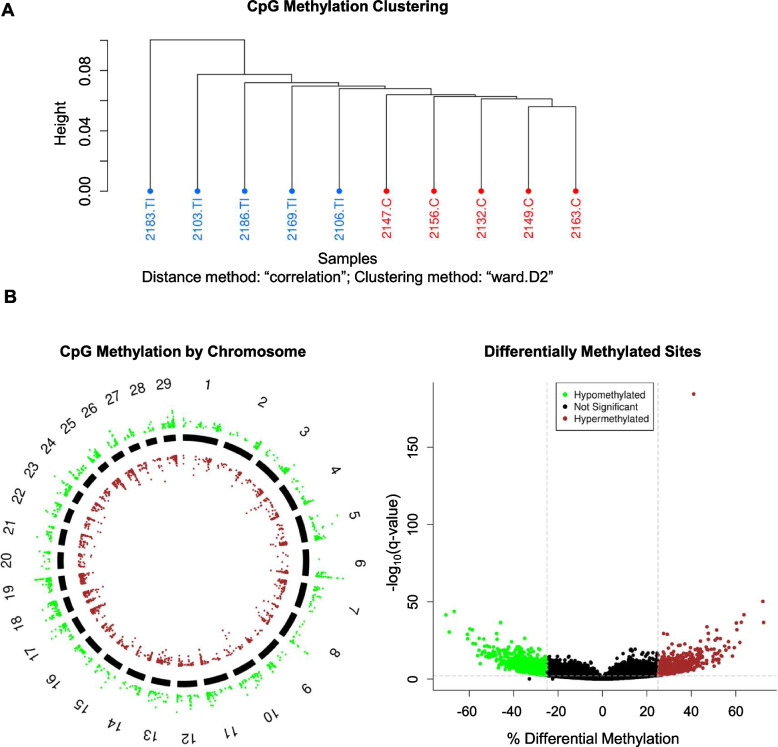
Differential methylation of CpG sites in TI heifers. The CpG sites are defined as sites containing a ‘CCGG’ motif within the genome. **A** Hierarchical clustering of sample methylation profiles based using 1—Pearson’s correlation distance. Control heifers are denoted in red and heifer ID numbers are followed by ‘.C’. Transiently infected TI heifers are denoted in blue and animal ID numbers are followed by ‘.TI’. **B** Visualization of differentially methylated CpG sites identified in TI heifers compared to controls by chromosome. Each data point represents a differentially methylated CpG site in respect to its location on the chromosome. Hypermethylated CpG sites are denoted in green and are positioned outside of the solid chromosome bars. Hypomethylated CpG sites are denoted in red and are positioned within the solid chromosome bars. **C** Visualization of the hypermethylated and hypomethylated CpG sites identified in TI cattle compared to controls. The –log10(q-value) Y axis threshold was set at 2 and denoted as a horizontal, grey dashed line. The % differential methylation X axis thresholds were set at − 25 and 25 and are denoted as vertical, grey dashed lines. Hypermethylated CpG sites are denoted in green and hypomethylated CpG sites are denoted in red

Whole blood was collected from 4-month-old heifers and processed to extract DNA from WBCs. These DNA samples were subjected to classic RRBS to determine CpG methylation status. The epigenomes of 5 TI heifers were compared to the epigenomes of 5 control heifers. The average rate of bisulfite conversion for all samples was 99.2% for non-CpG sites and 99.4% for known DNA spike-ins, which serve as a quantifiable control. Within the control group, 32.74% of bisulfite converted sequences aligned uniquely to the most recent reference genome and 51.02% aligned ambiguously. Within the TI group, 33.14% of bisulfite converted reads aligned uniquely and 50.16% aligned ambiguously to the reference genome. The average CpG coverage was 8X. Phred scores were above 30 at all base positions, indicating the potential for an incorrectly labeled nucleotide to be less than or equal to 1 in 1000 (99.9% accuracy). Hierarchical clustering of sample methylation profiles using 1 - Pearson’s correlation distance demonstrated overall similarity between control and TI heifers (Fig. 2A).

Identified differentially methylated CpG sites DMSs had a differential methylation (meth.diff) of greater than 25% and a false discovery rate of less than 0.01, per the calculateMethDiff() default. The meth.diff values were identified through logistic regression that compared the fraction of methylation in TI heifers to that of control heifers at a specific CpG site [26]. A total of 3,047 DMS were identified throughout the genomes of TI heifers when compared to controls (Fig. 2B). Of the DMSs, 1,698 sites were hypomethylated (56%; meth.diff > 25) and 1,349 sites were hypermethylated (44%; meth.diff < − 25; Fig. 2C).

Once identified, DMSs were annotated according to which gene body the site was contained within. The DMSs were then categorized based on their location within the gene body according to the reference genome obtained from the UCSC, 40% of DMSs were found within introns, 36% within intergenic regions, 13% in exons, and 11% within promoters. A total of 1,562 genes contained a single DMS, while 518 genes were identified to contain multiple DMSs (Table 1).

**Table 1:** Genes containing single or multiple differentially methylated CpG sites in TI heifers

Genes Containing	# of DMS	Adj. Q Value	Meth.Diff
1,562	1	4.28e^-^^185^ – 6.05e^-^^3^	−69.01 – 72.20
326	2	2.55e^-^^42^ – 5.55e^-^^3^	−58.35 – 72.58
106	3	3.29e^-^^30^ – 4.81e^-^^3^	−54.47 – 55.54
34	4	1.10e^-^^19^ – 2.81e^-^^4^	−52.54 – 47.57
17	5	1.84e^-^^37^ – 1.61e^-^^3^	−42.59 – 56.91
9	6	1.91e^-^^37^ – 1.57e^-^^3^	−41.23 – 62.53
8	7	3.30e^-^^42^ – 1.71e^-^^3^	−70.50 – 51.93
2	8	1.29e^-^^22^ – 1.46e^-^^5^	25.85–44.01
4	9	2.99e^-^^19^ – 5.73e^-^^4^	−51.29 – 39.33
5	10	1.73e^-^^21^ – 5.22e^-^^5^	−47.84 – 37.85
5	11	1.29e^-^^21^ – 9.07e^-^^4^	−50.97 – 41.51
1	13	3.17e^-^^16^ – 1.62e^-^^5^	−25.90 – 37.66
1	14	4.36e^-^^27^ – 8.17e^-^^4^	−55.56 – 51.62

Column 1 contains the quantification of genes containing the corresponding number of differentially methylated sites (DMSs) as listed in column 2. The ranges of adjusted *q*-value and percent of differential methylation (meth.diff) for the total number of genes in column 1 are listed in column 3 and 4

To identify differentially methylated CpG islands and promoters, differential methylation was required to be greater than 15% and have a false discovery rate of less than 0.01. Both CpG islands and promoter regions were identified using the most recent bovine reference genome (bostau9, 2018) from the UCSC. The meth.diff threshold was decreased when identifying regions to preserve potential true positives within the small sample size. Within TI heifers, 84 CpG islands were differentially methylated. Of the CpG Islands, 45 were hypomethylated (54%) and 39 were hypermethylated (46%). The TI heifers also exhibited differential methylation of 106 promoters, 62 of which were hypomethylated (58%) and 44 of which were hypermethylated (42%).

**Fig. 3:**
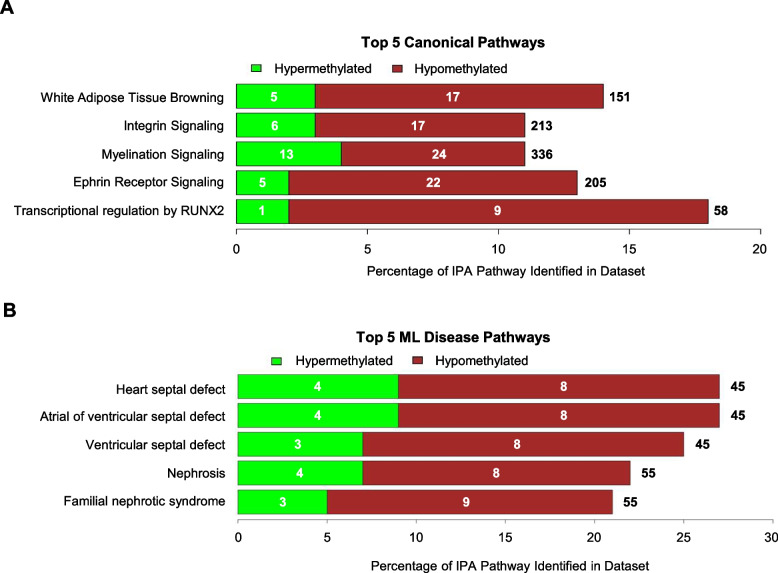
IPA predicted impacts on TI cattle through utilization of differentially methylated CpG sites. Top 5 canonical pathways and machine learning (ML) disease pathways as determined by IPA. Green denotes hypermethylation while red denotes hypomethylation. The total number of genes included in the IPA pathway is denoted to the right of each stacked bar. The number of hypo- or hyper- methylated genes identified in the dataset are denoted within each stacked bar. **A** Canonical pathways were sorted according to the absolute value of the predictive z-score determined by IPA. **B** Disease pathways were sorted according to the -log(p-value) determined by IPA

The DMSs identified during differential analysis of TI and control heifer epigenomes were further examined using IPA software (Qiagen, Germantown, MD, USA). The DMRs identified were not investigated using IPA software due to decreased quantity; the DMRs were investigated manually in accordance with DMSs pathway analysis. The IPA software identified which canonical pathways and disease pathways were most likely to be altered in the TI heifers compared to controls (Supplementary Fig. 1 and 2). Pathways identified by IPA list the number of genes identified within the dataset over the number of genes within the IPA pathway database. Canonical pathways in the IPA software are curated to the current understanding of biological functions. Pathways identified in IPA were sorted according to the absolute value of the z-score, from largest to smallest. A positive z-score indicates activation of the pathway, while a negative z-score indicates inhibition. The canonical pathways with the top highest z-scores include White Adipose Tissue Browning Pathway (3.13), Integrin Signaling (2.82), Myelination Signaling Pathway (2.8), Ephrin Signaling Pathway (2.67), and Transcriptional Regulation by RUNX2 (2.6; Fig. 3A). The IPA software includes pathways containing genes that are associated with specific diseases that have been generated by machine learning software. The top 5 ML disease pathways identified by IPA altered in TI heifers with the most significant p-value included heart septal defect (5.5), atrial or ventricular septal defect (5.5), ventricular septal defect (4.7), nephrosis (4.54), and familial nephrotic syndrome (4.54; Fig. 3B).

Several genes contained multiple sites of differential methylation within their promoter regions (Table 2). Genes are listed in the following format: *GENENAME* (methylation status; number of DMS with denoted methylated status). These genes include *BREH1* (hypermethylated × 4)*, ABCA9* (hypermethylated × 2)*, RIPOR2* (hypermethylated × 2)*, BCKDK* (hypermethylated × 2)*, LOC613282* (hypermethylated × 2)*, LOC104974744* (hypermethylated × 2)*, PODNL1* (hypomethylated × 5)*, ILVBL* (hypomethylated × 4)*, LOC112448525* (hypomethylated × 4)*, ASZ1* (hypomethylated × 2)*, SORBS1* (hypomethylated × 2)*, RUSC1* (hypomethylated × 2)*,* and *LOC112444767* (hypomethylated × 2)*.*

**Table 2:** Genes containing multiple differentially methylated CpG sites within promoter regions in TI heifers

Gene Name	Methylation Status	# of CpG Sites
*BREH1*	Hyper	4
*ABCA9*	Hyper	2
*RIPOR3*	Hyper	2
*BCKDK*	Hyper	2
*LOC613282*	Hyper	2
*LOC104974744*	Hyper	2
*ASZ1*	Hypo	5
*SORBS1*	Hypo	4
*RUSC1*	Hypo	2
*LOC112444767*	Hypo	2

Listing of genes containing multiple differentially methylated CpG sites within a promoter region in TI heifers. Information is presented in each row corresponds to the gene name, whether the promoter region within the gene contained hyper- or hypo- methylation, and the number of differentially methylated CpG sites contained within the promoter region

### Comparison of WBC methylation in TI heifers at birth and 4 months of age

The list of genes containing DMSs identified in the DNA of WBCs from TI heifers at 4 months of age was compared to those identified in the same TI heifers at birth [19]. At birth, 2,349 DMSs were identified within a total of 1,563 genes. Of these genes, 503 were identified to contain DMSs at 4 months of age. After removal of genes containing both a hypermethylated and hypomethylated DMS, 205 CpG sites were identified to contain the same differential methylation status in comparison to controls at birth and 4 months of age (S file 1 ‘d0 TI vs 4 moTI – DMS’). At birth and 4 months of age, 5 genes contained differentially methylated promoters, and 10 genes contained a differentially methylated CpG island. Genes that contained differentially methylated promoters at birth and 4 months of age include *UBXN11, MMP9, VWA3B, LOC520626,* and *LOC112449328* (Table 3)*.* Genes containing a differentially methylated CpG island include *EIF2S2, CACNG1, CELA3B, RAB20, UBAC1, LOC107131849, LOC101904842,* and *LOC112448374* (Table 3).

**Table 3:** Comparison of differential methylation in TI heifers at birth and 4 months of age

**Promoters**		
	**Methylation Status**	
**Gene Symbol**	**Birth**	**4 M.O.A.**
*MMP9*	Hypo	Hypo
*UBXN11*	Hyper	Hypo
*VWA3B*	Hypo	Hyper
*LOC520626*	Hypo	Hypo
*LOC112449328*	Hypo	Hypo
**CpG Islands**		
	**Methylation Status**	
**Gene Symbol**	**Birth**	**4 M.O.A.**
*CACNG1*	Hypo	Hyper
*CELA3B*	Hyper	Hyper
*EIF2S2*	Hyper	Hypo
*RAB20*	Hypo	Hypo
*UBAC1*	Hypo	Hypo
*LOC107131849*	Hypo	Hypo
*LOC101904842*	Hyper	Hyper
*LOC101907146*	Hyper	Hypo
*LOC112448374*	Hyper/Hypo	Hypo x2

Listing of genes containing a differentially methylated promoter or CpG island at birth and 4 months of age (M.O.A.) in TI heifers. The list of genes containing differential methylation in TI calves compared to controls at 4 M.O.A. was generated herein. The list of genes containing differential methylation in TI calves compared to controls at birth was generated in [19]. The methylation status (hypermethylated or hypomethylated) is denoted at birth and 4 M.O.A. Genes containing more than one site of differential methylation list the methylation status of both sites. If multiple sites within a promoter or island have the same methylation status, xN is used to denote multiple sites, as in (x2)

**Fig. 4:**
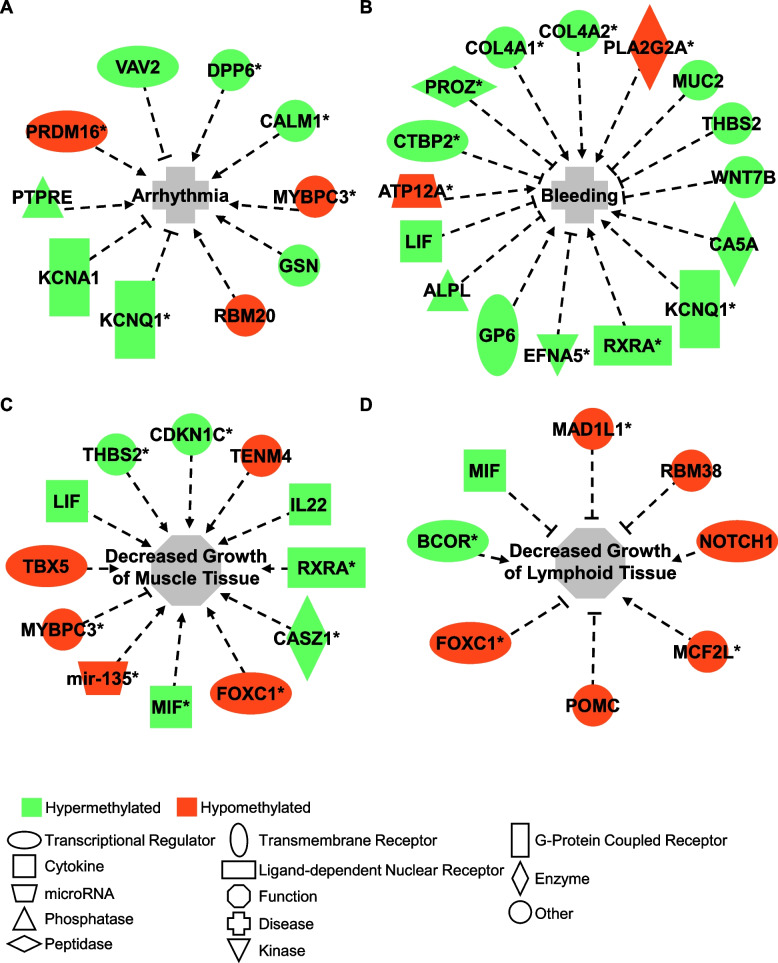
IPA predicts increased risk of arrhythmia and bleeding, decreased growth of muscle and lymphoid tissue. Modified IPA figures depict genes containing differential methylation that contribute to central diseases or altered functions. **A** Genes associated with arrhythmia. **B** Genes associated with increased bleeding. **C** Genes associated with decreased growth of muscle tissue. **D** Genes associated with decreased growth of lymphoid tissue. Green indicates hypermethylation, red indicates hypomethylation. Dashed lines ending with a flat line end indicate inhibition. Dashed lines ending with an arrow indicate contribution to the central disease or altered state. Asterisks following a gene name indicate that multiple identifiers in the dataset map to a single gene or chemical in the Global Molecular Network

Genes that were identified to contain differential methylation at birth and 4 months of age in TI heifers were extracted from their retrospective datasets and utilized to perform a comparative analysis in IPA. Canonical pathways identified to be altered in TI heifers at birth and 4 months of age include epithelial adherens junction signaling (activated), atherosclerosis signaling (activated), Signaling by Rho family GTPases (activated), cardiac conduction (inhibited), and L1CAM interactions (inhibited). Using IPA, it was predicted that TI heifers experience decreased growth of lymphoid tissue (Fig. 4A), decreased growth of muscle tissue (Fig. 4B), as well as increased differentiation of keratinocytes, development of hematopoietic progenitor cells, differentiation of helper T lymphocytes, and maturation of T lymphocytes. It was also predicted through IPA that TI heifers would suffer from increased bleeding and longer clotting times following injury (Fig. 4C). Toxicological pathways identified indicate increased levels of alkaline phosphatase, risk of arrhythmia (Fig. 4D), and proliferation of cardiomyocytes. The IPA software utilizes input data to infer and identify upstream regulators that, if altered, may explain the input data. Upstream regulators identified by IPA include *FGFR1, FGF2, DICER1, FOXC2, INHBA, SOX2, SOX3, SOX9, KLF5, LIF, PTH, cyclic AMP, GLI1,* and *IL6,* among others (S file 2 ‘d0 TI vs 4 moTI – Upstream Regulators’).

### Comparison of WBC methylation in TI and PI heifers

The list of genes containing DMSs in TI heifers at 4 months of age was also compared to the list of genes containing DMSs in PI heifers at 4 months of age [24]. The 8,367 DMSs identified in PI heifers were found on a total of 3,812 genes. When compared, 1,098 genes containing at least 1 DMSs were found in both TI and PI heifers at 4 months of age. After removal of genes containing both a hypermethylated and hypomethylated DMS, 465 CpG sites were identified to contain the same differential methylation status in comparison to controls in TI and PI heifers (S file 3 ‘4 moTI vs 4 moPI – DMS’). Comparison of genes containing differentially methylated promoters revealed 18 genes in common between TI and PI heifers (Table 4). Comparison of differentially methylated CpG islands yielded 34 genes in common between TI and PI heifers at 4 months of age (Table 4).

**Table 4:** Comparison of differential methylation in TI and PI heifers at 4 months of age

**Promoters**		
	**Methylation Status**	
**Gene Symbol**	**TI**	**PI**
*ABCA2*	Hypo	Hypo
*ABCA9*	Hypo x2	Hyper x2
*GBP4*	Hypo	Hypo
*KIFC3*	Hyper	Hyper
*LOC112444931*	Hypo	Hypo
*LOC112447841*	Hypo	Hypo
*LOC112448077*	Hypo	Hypo
*LOC112449328*	Hypo	Hypo
*LOC112449597*	Hyper	Hypo
*LOC522763*	Hypo	Hypo
*MMP9*	Hypo	Hypo
*NEDD4L*	Hypo	Hyper
*OR4C1J*	Hyper	Hyper
*P2RY11*	Hyper	Hyper
*PKNOX2*	Hypo	Hypo
*SPNS2*	Hypo	Hypo
*TKDP4*	Hypo	Hypo
*TNNC1*	Hypo	Hypo
**CpG Islands**		
	**Methylation Status**	
**Gene Symbol**	**TI**	**PI**
*BOC*	Hyper	Hyper
*CACNG1*	Hyper	Hypo
*DTX2*	Hypo	Hypo
*EIF2S2*	Hypo	Hypo
*FOXS1*	Hyper	Hyper
*GATA3*	Hypo	Hypo
*GP9*	Hypo	Hypo
*IGSF22*	Hyper	Hyper
*INPP5 A*	Hypo	Hypo
*IRF4*	hypo	Hypo
*LOC101906717*	Hyper	Hypo
*LOC101907140*	Hypo	Hypo
*LOC104972957*	Hypo	Hypo
*LOC104976302*	Hypo	Hypo
*LOC107131765*	Hypo/Hyper	Hyper
*LOC107131849*	Hypo	Hypo
*LOC107132967*	Hypo	Hypo
*LOC112443431*	Hypo/Hyper	Hypo
*LOC112443852*	Hyper	Hyper
*LOC112444931*	Hypo	Hypo
*LOC112448161*	Hyper	Hyper
*LOC112448374*	Hypo	Hypo
*LOC112449090*	Hypo	Hypo
*MEF2A*	Hypo	Hypo
*MX1*	Hyper	Hyper
*PES1*	Hypo	Hypo
*PITPNA*	Hypo	Hypo
*PRR18*	Hyper	Hypo
*SMYD2*	Hyper	Hyper
*SNTG2*	Hypo	Hypo
*SPTBN4*	Hyper	Hyper
*TMEM91*	Hyper	Hyper
*TRMU*	Hyper	Hyper
*UBE2T*	Hypo	Hypo

Listing of genes containing a differentially methylated promoter or CpG island in either TI or PI heifers at 4 months of age. The list of genes containing differential methylation in TI calves compared to controls was generated herein. The list of genes containing differential methylation in PI calves compared to controls was generated in [24]. The methylation status (hypermethylated or hypomethylated) is denoted in TI calves and PI calves. Genes containing more than one site of differential methylation list the methylation status of both sites. If multiple sites within a promoter or island have the same methylation status, xN is used to denote multiple sites, as in (x2)

The list of genes containing differential methylation at single or multiple CpG sites in TI heifers at 4 months of age was compared to those identified in PI heifers at 4 months of age. Canonical pathways identified by IPA include the role of osteoclasts in rheumatoid arthritis signaling, GP6 signaling, GPVI-mediated activation cascade, wound healing signaling, Rho GTPase cycle, signaling by Rho family GTPases, and G-protein coupled receptor signaling, and others (S file 4 ‘4 moTI vs 4 moPI – Canonical Pathways). Upstream regulators predicted to be altered include *EGLN2, BDNF, NOTCH1, SOX2, BMPR1A, SAMD8, BACH2, SPP1,* and *LAMP2.* A full list of upstream regulators predicted to be altered in both TI and PI heifers can be found in S file 5 ‘4 moTI vs 4 moPI – Upstream Regulators.’

### Complete blood counts

Whole blood was collected from the jugular vein of control and TI heifers at 4 months of age. Blood samples were then analyzed for CBCs (Table 5). The percentage of lymphocytes was higher in TI heifers at 4 months of age compared to controls (68.44 ± 1.79% vs 73.25 ± 1.41%; p-value ≤ 0.05). Other CBC measurements that were not different between control and TI heifers include the absolute basophil count, absolute eosinophil count, absolute neutrophil count, absolute monocyte count, monocyte percentage, absolute red blood cell count, hemoglobin, hematocrit, red cell distribution width, mean corpuscular volume, mean corpuscular hemoglobin, mean corpuscular hemoglobin concentration, absolute white blood cell count, absolute lymphocyte count, absolute platelet count, and mean platelet volume.

**Table 5:** Increased lymphocyte percentage in TI heifers

Complete Blood Count Parameter	Control Average ± SEM	TI Average ± SEM	Unit	*p*-value
Basophil Count †	0.05 ± 0.01	0.04 ± 0.01	10^9^/L	0.98
Eosinophil Count	0.07 ± 0.01	0.09 ± 0.02	10^9^/L	0.27
Neutrophil Count	2.89 ± 0.27	2.31 ± 0.36	10^9^/L	0.21
Monocyte Count	0.38 ± 0.04	0.35 ± 0.06	10^9^/L	0.69
Monocyte Percentage	3.53 ± 0.41	3.2 ± 0.39	%	0.57
Red Blood Cell Count	12.17 ± 0.22	12.03 ± 0.43	10^12^/L	0.78
Hemoglobin	14.28 ± 0.23	13.73 ± 0.47	g/dL	0.31
Hematocrit	41.33 ± 0.72	40.12 ± 1.33	%	0.44
Red Cell Distribution Width	22.96 ± 0.34	23.42 ± 0.45	%	0.68
Mean Corpuscular Volume	33.99 ± 0.44	33.41 ± 0.6	fL	0.44
Mean Corpuscular Hemoglobin	11.76 ± 0.15	11.44 ± 0.24	pg	0.28
Mean Corpuscular Hemoglobin Concentration	34.59 ± 0.16	34.22 ± 0.16	g/dL	0.12
White Blood Cell Count	10.68 ± 0.46	10.09 ± 1.28	10^9^/L	0.67
Lymphocyte Count	7.28 ± 0.3	7.28 ± 0.89	10^9^/L	0.99
Lymphocyte Percentage *	68.44 ± 1.79	73.25 ± 1.41	%	0.05
Platelet Count	462.8 ± 51.53	332.5 ± 78.92	10^9^/L	0.18
Mean Platelet Volume	4.93 ± 0.06	4.74 ± 0.12	fL	0.18

Quantification of the average complete blood count values in TI and control heifers at 4 months of age. Data were first assessed using the Shapiro-Wilk normality test. Outliers were removed prior to significance testing. The dagger (†) was utilized to indicate which cell measures contained outliers that were removed. Data that were normally distributed were compared using an unpaired, parametric Student’s T test. Welch’s correction was applied to all T test comparisons due to unequal sample populations (Control n = 12, TI n = 11; prior to outlier removal). Data that was not normally distributed were compared using a nonparametric Mann-Whitney test. Data are displayed as mean ± standard error of the mean (SEM). Asterisks indicate significant differences between groups, * *P* ≤ 0.05

### Flow cytometry

**Fig. 5:**
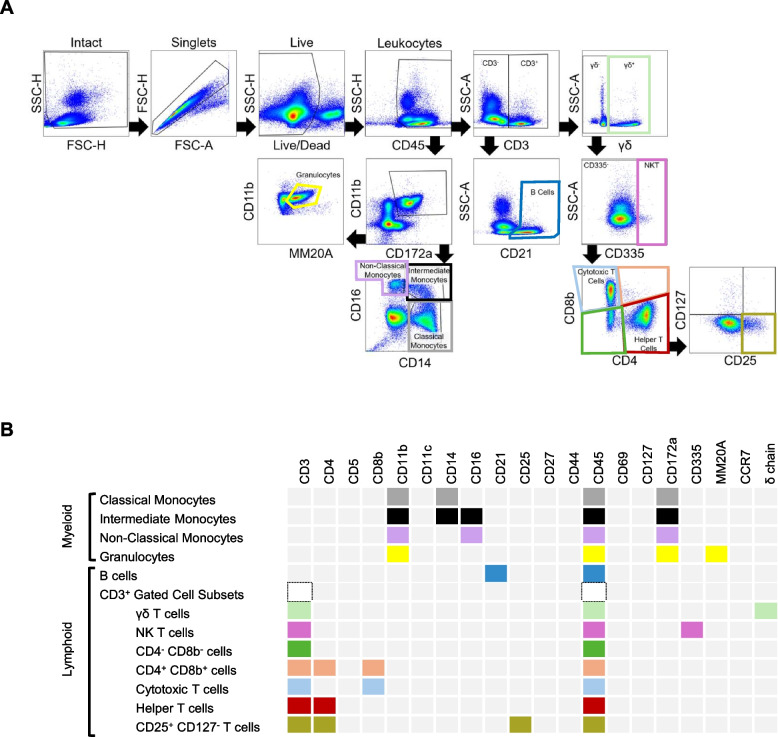
Markers utilized to identify immune cell populations. **A** A schematic depicting the fluorescent markers used to identify various immune cell populations through flow cytometry. Colors used here correspond to those utilized in Table 4. Boxes shaded in light grey were not identified in populations, while boxes shaded in various colors (including white) indicate positive fluorescence in a cellular population. **B** Schematic representation of the gating strategy used to identify various immune cell populations. Colors here correspond to colors representing various populations in Table 6

Peripheral WBCs were isolated from whole blood collected from the jugular vein of control and TI heifers at 4 months of age. Flow cytometry was utilized to investigate the impact of fetal TI on postnatal immune cell populations (Fig. 5 and Table 6). Gating was performed to remove debris, doublet, and dead cells. General populations of cells were first compared based on their frequency within total leukocytes (CD45^+^ cells). Subpopulations of T cells were also compared according to their frequency within total T cells (CD3^+^). As a subset of Helper T cells, the CD25^+^/CD127^−^ population frequency within CD4^+^ was compared to mitigate external bias.

**Table 6:**
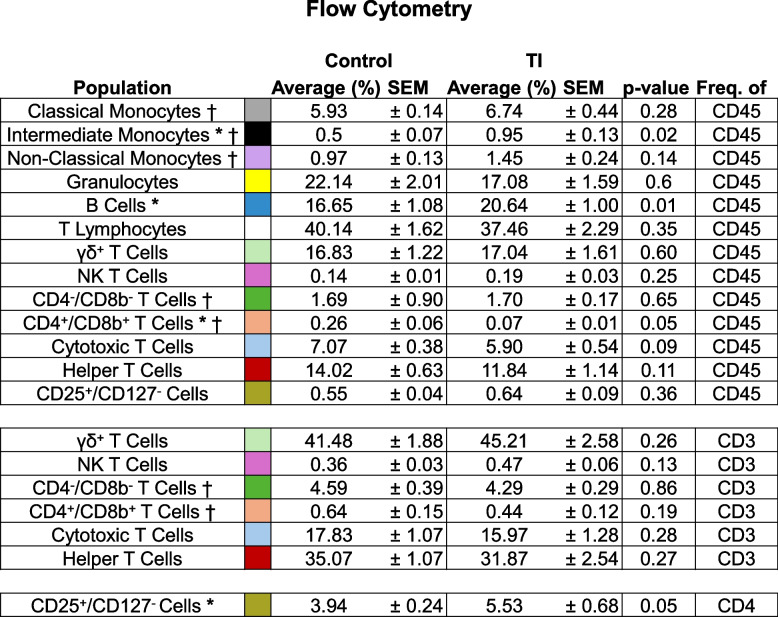
TI heifers display eleva﻿ted intermediate monocytes and B cells, but decreased CD4^+^/CD8b^+^ and CD25^+^/CD127^-^ T cells

Quantification of cell population frequency in relation to the indicated parent population. Shading color corresponds to cell population markers in Figure 3. Data was first assessed using the Shapiro-Wilk normality test. Outliers were removed prior to significance testing. The dagger (†) was utilized to indicate which cell measures contained outliers that were removed. Data that were normally distributed were compared using an unpaired, parametric Student’s T test. Welch’s correction was applied to all T test comparisons due to unequal sample populations (Control n = 12, TI n = 11; prior to outlier removal). Data that was not normally distributed were compared using a nonparametric Mann-Whitney test. Data are displayed as mean ± standard error of the mean (SEM). Asterisks indicate significant differences between groups, * *P* ≤ 0.05

When comparing frequency of CD45^+^ cells, intermediate monocytes (0.5 ± 0.14% vs 0.95 ± 0.44%; p-value ≤ 0.05) and B cells (16.65 ± 1.08% vs 20.64 ± 1%; p-value ≤ 0.01) were higher in TI heifers than in controls. Identified T cells positive for both CD4^+^ and CD8^+^ were decreased in TI cattle (0.26 ± 0.06% vs 0.07 ± 0.01%; p-value < 0.05). When CD25^+^/CD127^−^ cells were compared using frequency of CD4^+^ cells, they were found to be increased in TI heifers (3.94 ± 0.24% vs 5.53 ± 0.68%; p-value < 0.05). Frequency of classical monocytes, non-classical monocytes, granulocytes, T lymphocytes, gamma delta (γδ^+^) T cells, natural killer (NK) T cells, CD4^−^/CD8^−^ T cells, cytotoxic T cells, helper T cells, and CD25^+^/CD127^−^ T cells within total leukocytes (CD45^+^) were not different between groups. Subpopulations of T cells were not different when frequency of CD3^+^ cells was compared.

## Discussion

Billions of dollars in potential profit are lost each year due to BVDV infections [1, 2]. Currently, TI cattle generated through fetal infection cannot be distinguished from uninfected cattle and may induce additional loss of potential profit through decreased weight gain and increased susceptibility to severe morbidity [16, 18, 19]. The TI heifers in this study were generated through gestational infection with ncp BVDV type 2. Differentially methylated genes identified in TI heifers are associated with inflammation, ROS production, and insulin secretion. These findings are supported by increased concentrations of inflammatory markers found in TI heifers [18]. The CBC values identified in TI and control heifers do not often fall within reference ranges for cattle, likely due to differences in age, breed, and lactation status [30]. The CBC values identified in TI calves were not different from those of controls, except for the case of lymphocyte percentage, which is corroborated by flow cytometry. Spectral flow cytometry maintains increased resolution and sensitivity in comparison to CBC analysis, allowing for the observation of differences in lymphocyte subsets. As such, flow cytometry revealed that TI heifers had increased cellular populations of B cells and CD25^+^/CD127^−^ T cells. By analyzing individual monocyte subpopulations, rather than monocytes as a sum, intermediate monocytes were observed to be increased in TI heifers compared to controls. The TI heifers were also found to have decreased cellular populations of CD4^+^/CD8b^+^ T cells. This study sheds light on potential pathologies associated with TI heifers and provides foundational knowledge for the development of management strategies to mitigate profit loss.

Differential methylation of genes associated with observed clinical pathologies in PI cattle has been described [24]. Moreover, differential methylation has been identified in TI heifers at birth [19]. Analysis of WBC DNA of TI and control heifer calves at birth revealed 2,349 DMSs with the potential to influence growth, development, and the immune system [19]. A study by Munoz-Zanzi et al. [16] describes calves positive for BVDV antibodies at birth, termed congenitally infected (CI; synonymous with TI), as having 2.3 times the risk of a severe morbid episode in comparison to non-CI calves. A severe morbid episode is defined as an illness requiring 3 or more days of treatment followed by recovery, or death without record of previous [16]. Of the 41 CI calves, 9 experienced a severe morbid episode [16].

Many of the pathways identified by IPA pose the potential to increase the risk of cancer development in postnatal TI calves. Activation of integrin signaling is often associated with tumorigenesis and tumor invasion [31–33], as is ephrin signaling [34], GTPase signaling [35], and the predicted activated upstream regulator *SOX9* [36]. If a TI calf developed cancer, it is possible that other epigenetic alterations could enhance the development and metastasis of cancer, as is demonstrated by predicted activation of the transcriptional regulation by RUNX2 pathway which can promote tumor growth and metastasis [37]. Increases in cardiac defects or nephrosis, as predicted by IPA, may ultimately lead to loss of profit due to premature death of TI calves. However, the impact would depend on the severity of the defect or progression of disease.

At 4 months of age, lymphocyte percentage was elevated in TI heifers compared to controls. However, the average absolute lymphocyte count was identical between groups. The highest lymphocyte percentage identified in the TI group was 80.8%, which is within the normal range for cattle under the age of 2 years [38]. Using spectral cytometry, TI heifers were identified to have increased percentages of B cells and CD25^+^/CD127^−^ T cells. A larger circulating B cell population in TI calves compared to controls could be explained by fetal exposure to BVDV, causing an expansion in circulating memory B cells [39].

The CD4^+^ subpopulation of cells defined by expression of CD25^+^ and lack of CD127^−^ expression is defined as T regulatory cells in humans. However, these cells do not exert immunosuppressive activity in the bovine [40]. Little is known about CD4^+^ CD25^+^/CD127^−^ T cells in bovine. The CD4^+^/CD8b^+^ T cell population was decreased in TI heifers compared to controls. It has been speculated that the CD4^+^/CD8b^+^ T cell population may be an uncharacterized population of peripheral T helper cells, as an increase in CD4^+^/CD8^+^ T cells was observed in accordance with B cell proliferation following vaccination for foot and mouth disease in cattle [41]. In humans, an increased amount of CD4^+^/CD8^+^ T cells has been observed in cases of viral infection and chronic disease (reviewed in [42]). This observation is in accordance with observed increases of CD4^+^/CD8^+^ T cells in PI heifers, which experience chronic BVDV infection [24]. However, neither TI nor control calves displayed any clinical signs of illness at the time of sample collection. Additional research characterizing this cell population is necessary to determine the global impact of their elevation.

Flow cytometry also revealed increased intermediate monocytes in TI heifers compared to controls. Several genes known to be enriched in intermediate monocytes were found to contain hypomethylated CpG sites in TI heifers, including *CCL5, C1QA, IRF4, GBP4,* and *MAFB* [43]*.* Additionally, *GBP4* was also found to contain hypomethylation within a promoter region and *IRF4* contained hypomethylation within a CpG island. Increased expression of *IRF4* and *GBP4*, which is associated with hypomethylation, could push TI calves towards T cell exhaustion and an inability to appropriately respond to infection [44, 45]. Intermediate monocytes in the bovine express the highest amount of MHCII on their cell surface, as well as produce the largest amount of reactive oxygen species and pro-inflammatory cytokines such as CXCL8, CXCL1, IL1B and TNF [46]. An increase in absolute count of intermediate monocytes in TI calves may indicate stimulation of the immune system prior to data collection at 4 months of age, as treatment of classical monocytes in vitro with IFNγ resulted in expansion of the intermediate monocyte population [46]. However, no clinical signs were noted in TI heifers at the time of blood collection.

### A cascade of aberrant methylation

The DNA methyltransferase 3 (DNMT3) family contains DNMT3A, DNMT3B, and DNMT3L [47, 48]. The DNMT3L protein is catalytically inactive regulatory factor for DNMT3A and DNMT3B [49]. Interestingly, *DNMT3L* contained hypermethylated DMSs at birth in TI heifers, but was not differentially methylated at 4 months of age. The DNMT3L protein is highly expressed during gametogenesis [50]. Mice lacking DNMT3L produce oocytes with defects in maternal imprinting, leading to death of offspring [49, 50]. The maternal imprinting process is dependent on oocyte growth in bovine [51] and only primordial follicles are present in pre-pubertal heifers. Differential methylation of *DNMT3L* may impact fertility of these TI calves following pubertal development. The DNMT3L protein is known to enhance the activity of DNMT3A and DNMT3B [52]. As such, it is possible that hypermethylation of *DNMT3L*, associated with decreased expression, could lead to a decrease in DNMT3A and DNMT3B activity and de novo methylation in the perinatal period.

Expression of DNA methyltransferases is markedly decreased in adult tissues and decreases overall as organisms age [53]. A study by Zhenhua Li et al. recently demonstrated that overexpression of DNMT3A in rats leads to variation of gene expression, specifically within the brain and testis [54]. It is possible that the scenario is similar in the present TI calf study; hypomethylation of *DNMT3A* in TI calves could lead to overexpression and alteration of methylation and expression of other genes. This suggestion is supported by the identification of hypomethylation within *DNMT3A* and *DNMT3B* in PI heifers, who are infected earlier in gestation and present with more clinical abnormalities [24].

### Differential methylation of genes associated with inflammation and ROS production

Inflammation and ROS production are linked processes with the potential to induce damage if not regulated appropriately. Increased inflammation can lead to increased ROS production and vice versa. Typically, this self-amplifying cycle is mediated by cellular antioxidant systems, but these systems can be overwhelmed and lead to oxidative stress. Analysis of DMSs by IPA predicted an increase in oxidative stress, citing hypermethylation of CpG sites within genes encoding *GSTA3, SOD1, MAFK,* and *PGC1A.*

During a feedlot trial, TI calves were found to exhibit signs of increased inflammation including elevated levels of oxidized glutathione and plasma ceruloplasmin concentrations [18]. No clinical signs of illness were observed in the heifers during the trial. If TI calves maintain a higher base level of inflammation, it is possible that immune activation would lead to oxidative stress. The CCL5 chemokine, containing a hypomethylated DMS in TI calves at 4 months of age, has been implicated in several inflammatory diseases in humans (reviewed in [55]) and is transcriptionally regulated by KLF13 [56]. The KLF13 gene also contained at least one hypomethylated CpG site in TI calves at birth and at 4 months of age [19]. Similarly, CCL24 is associated with inflammatory or fibrotic disease and contained a hypomethylated CpG site in TI calves [57, 58]. Another gene, *LIF*, which is known to have immunomodulatory effects [19], contained hypermethylated CpG sites; 1 site was identified within the gene at birth and 3 sites were identified at 4 months of age in TI heifers. The IPA software also predicted LIF to be an activated upstream regulator. Human T regulatory cells are stimulated to proliferate in the presence of LIF, while Th17 cell proliferation is inhibited by LIF [59]. Dendritic and macrophage inflammatory responses are also mitigated by *LIF* expression [60, 61]. Hypomethylation of inflammatory-associated genes in TI heifers along with hypermethylation of immunosuppressive genes may indicate chronic inflammation.

Immune stimulated inflammation can also lead to the upregulation of IL17 receptors [62]. The IL17 receptor A (IL17RA) was found to contain a hypomethylated CpG site in TI heifers and is one of five IL17 receptors and is required for the proinflammatory activity of IL17A [63]. The activation of IL17RA induces inflammation through chemokine and cytokine expression, but also recruits neutrophils, activates T cells, and enhances migration of monocytes (reviewed in [64–66]. Activation of the immune response leads to proliferation and activation of phagocytic cells that are known to produce high amounts of ROS through respiratory burst [67, 68]. Specifically, lipopolysaccharide stimulated inflammation can lead to the release of redox molecules from both macrophages and monocytes [69]. Phagocytic cells produce ROS through NADPH oxidases, specifically through NOX2 [67, 68]. A cytosolic subunit encoded by *Rac1* is required for activation of NOX2 and was identified to contain a hypomethylated CpG site in TI heifers. Intermediate monocytes were identified to be increased in TI heifers at 4 months of age. While the phagocytic activity of intermediate monocytes is lower than that of classical monocytes and higher than that of non-classical monocytes, they generate the highest amount of ROS in response to stimulation [46]. Together, these findings suggest that TI cattle may have higher risk of oxidative stress upon immune activation. Moreover, it has been noted that lipoprotein lipase (LPL) upregulates macrophage activation, and inflammation, in the presence of increased ROS [70]. The *LPL* gene was found to contain a hypomethylated CpG site in TI heifers, which may indicate increased expression and perpetuation of the inflammation-ROS cycle leading to oxidative stress.

A recent study described 66 CpG sites associated with markers of oxidative stress [71], 4 of which were identified to contain a differentially methylated CpG site in TI heifers. The genes *LRIG1, FAM20C,* and *EPHB1* contained a hypomethylated CpG site and *CBFA2T3* contained a hypermethylated CpG site*.* Additionally, the glutathione S-transferase alpha 3 gene, *GSTA3*, contained a hypermethylated CpG site in TI heifers and has been shown to increase ROS production when knocked down in cell culture [72]. The superoxide dismutase, *SOD1*, was found to contain a hypermethylated CpG site in TI heifers at 4 months of age. It has been described that a deficiency of SOD1 can lead to decreased weight at a young age, increased oxidative stress, and decreased DNA methylation in the prostate of mice [73]. While SOD1 is an important antioxidant enzyme, it has been suggested that other anti-oxidant systems can compensate for loss of SOD1 [74].

Hypomethylation of a CpG site within *NDOR1, SDHB,* and *GSTK1* suggests the need for increased expression of alternative antioxidant genes as the physiological systems attempt to mediate abnormal levels of ROS production [75–79]. However, the gene encoding MAFK contained a hypermethylated CpG site and is required for induction of antioxidant and detoxification enzymes [80]. The peroxisome proliferator-activated receptor γ coactivator 1-alpha, PCG1A, which is directly regulated by NFκB, was found to contain a hypermethylated CpG site in 4-month-old TI calves. During infection, an increase in NFκB would inversely lead to a decrease in PGC1A, which further leads to a transcriptional decrease of antioxidant genes [81]. Calves affected by fetal TI maintain differential methylation indicative of increased inflammation, ROS production, and a potentially stunted ability to manage ROS imbalances, which are likely to contribute to increased oxidative stress.

### Methylation and metabolic dysfunction

Inflammation and oxidative stress have also been implicated in metabolic dysfunction. Oxidative stress plays a role in downregulation of the transcription factor MAFA, which influences insulin secretion by pancreatic beta cells [82]. Interestingly, *MAFA* contained a hypermethylated CpG site in TI heifers, which may impair insulin secretion, as mice lacking MAFA display reduced expression of insulin in beta cells [83]. The gene encoding *PGC1A* contained a hypermethylated CpG site in TI heifers and is involved in redox balancing, inflammation, and metabolic disease (reviewed in [84]). Downregulation of PGC1A can enhance the inflammatory response, increase ROS production, and lead to insulin resistance and glucose intolerance [85].

In accordance with suggested metabolic dysfunction, hypermethylation of a CpG site within *DGKD, TRPC3,* and two CpG sites within *KCNB1* may contribute to abnormal insulin secretion. The diacylglycerol (DAG) kinase is responsible for the conversion of DAG into phosphatidic acid [86]. Elevated levels of DAG have been associated with the development of insulin resistance in type 2 diabetes (reviewed in [87]). Inhibition of *TRPC3,* whether through pharmacologic inhibition or knockout, results in decreased insulin secretion and glucose intolerance in mice [88]. The voltage-dependent potassium channel *KCNB1* is involved in exocytosis of insulin from pancreatic beta cells [89]. Additionally, the retinoid X receptor alpha (*RXRA*) was hypomethylated at 10 different CpG sites and hypermethylated at a single CpG site in TI heifers. One study described that RXRs act to inhibit insulin secretion in response to high glucose levels [90].

While IPA predicted overall activation of the White Adipose Tissue Browning pathway with a z-score of 3.13, it was predicted that there would be a decrease in mitochondrial biogenesis, differentiation of white adipocytes, and transdifferentiation of white adipose tissue to beige adipose tissue. The *ADCY* gene was identified by IPA to contain hypomethylation, but upon further investigation, it was actually the gene encoding ADCY6 which contained a hypomethylated CpG site in the gene body and a hypomethylated CpG island. Upregulation of ADCY6 is associated with increased hepatic glucose production, which can contribute to high blood glucose and overall glucose intolerance [91]. The gene encoding for positive regulatory domain containing 16, PRDM16, contained 4 hypomethylated CpG sites and a single hypermethylated CpG site. Due to decreased methylation within *PRDM16*, one would expect the potential for increased expression. As such, overexpression of PRDM16 would inhibit differentiation of white adipocytes and conversely encourage the formation of brown and beige adipocytes which expend energy as heat [92]. Brown and/or beige adipose tissue are not favorable in cattle, as expense of energy as heat decreases the average daily gain, which was observed in TI heifers [18]. The *DICER1* gene, which was predicted to be a possibly inhibited upstream regulator, is also known to impact weight gain. In fact, mice lacking DICER1 expression were found to maintain a lower body weight than controls [93]. Decreased average daily gain and decreased muscle growth, as predicted by IPA, translate to increased input costs and decreased profit return for cattle producers.

### Persistence of methylation in TI heifers

 Persistence of DNA methylation was observed in TI heifers through analysis of differential methylation at birth and 4 months of age. It is important to note that while DNA methylation is stable and heritable, this statement refers to the ability of such to remain throughout the cellular replication process. More recently it has been reported that DNA methylation does vary over time within individuals [94]. Specifically, DNA methylation decreases globally with age [53, 95], but simultaneously increases in CpG islands [96–99]. Because RRBS specifically identifies methylation at CpG sites, the increase from 2,349 DMSs at birth to 3,047 DMSs at 4 months of age demonstrates the site-specific increase in methylation, but not the global decrease in methylation.

Not only does the percentage of methylation change over time, but the methylation status of genes can change. For example, hormonal changes in puberty have been associated with changes in DNA methylation in humans [100]. More recently, even SARS-CoV- 2 infection has been noted to lead to variation in DNA methylation [100]. In theory, access to genetic material may be dependent upon the physiological need indicated by the organism. As TI heifers continue to develop, it is likely that the genes containing differential methylation will also change.

Variations in DNA methylation have been identified to play a role in inducing postnatal disease in organisms exposed to sub-optimal conditions in utero, which indicates some level of persistence epigenetic marks of DNA methylation. For example, differential methylation of genes corresponding to observed pathologies was identified in humans following prenatal exposure to malnutrition [101, 102]. The methylation status of DNA may also indicate the risk of occurrence or severity of disease, as in the case of type 2 diabetes and cardiovascular disease [103, 104]. It is possible that variations in DNA methylation in TI heifers indicate dysfunction of various physiological systems. Both at birth and 4 months of age, TI heifers were predicted to experience decreased growth of lymphoid tissue. This may support the claim that TI heifers mount an abnormal immune response, as lymphoid tissue is critical to appropriate immune response [105]. As this longitudinal study continues, variation in DNA methylation may prove critical in identification of cattle affected by late gestation BVDV infection.

### Commonalities between TI and PI heifers

It is evident that exposure to environmental stressors in utero can influence DNA methylation and there are time periods during gestation in which the fetus is especially susceptible to these factors. One study denotes that the changes in DNA methylation appear to be dependent upon sex of the fetus and gestational timing of exposure, and that exposure in late gestation resulted in fewer significant variations in DNA methylation of targeted genes [101]. All control, TI, and PI cattle utilized in this study were heifers, to eliminate the potential of sex differences. It is also well documented that the gestational timing of BVDV infection leads to vastly different outcomes for infected fetuses. The findings of differential methylation in TI and PI heifers agree with the concept that fetuses in early gestation are more ‘sensitive’ to DNA methylation variation than those in late gestation.

Many commonalities were identified in the variations of DNA methylation in TI and PI heifers. For example, *IL22* contained a hypermethylated CpG site in both TI and PI heifers. Interestingly, methylation of *IL22* increased in TI heifers from birth to 4 months of age (meth.diff = 26.22 at birth, 72.2 at 4 months) and even surpassed the level of hypermethylation in PI heifers at 4 months of age (meth.diff = 31.1 at 4 months). The IL22 signaling pathway was predicted to be inhibited by IPA, citing the identification of hypermethylation of a CpG site within *IL22* and *MAPK11.* The software predicted a decrease in negative feedback control of IL22 signaling and JNK. Additionally, mice lacking IL22 expression experience increased severity of colitis infections following chemical or bacterial induction [106–108]. On the other hand, macrophage migration inhibitor factor (MIF) contained a hypermethylated CpG site in TI and PI heifers. Mice lacking MIF were at least partially protected from the development of chemical induced colitis [109, 110]. Not all genes identified in TI and PI heifers are as contradictory as *IL22* and *MIF.*

Genes that are similarly methylated in TI and PI heifers may support the predisposition of PI cattle to bacterial infections and support the previous study denoting more severe illness in TI cattle [16]. Myxovirus resistance protein 1 (*MX1*) was found to contain 3 hypermethylated CpG sites in TI calves and 7 hypermethylated CpG sites in PI heifers. This protein acts as a cellular defense against viral infection. As such, a lack of MX1 has been shown to increase susceptibility of mice to influenza [111]. Mice without MPEG1, which contained a single hypermethylated CpG site in TI calves and two hypermethylated CpG sites in PI heifers, are also more susceptible to infection by inhibiting early transcription and replication of viral RNA [112, 113]. Lack of adequate MX1 and MPEG1 in TI and PI heifers may contribute to an immunocompromised state, which has at least been documented in PI cattle [114].

At 4 months of age, PI heifers display differential methylation of genes involved in blood coagulation [24]. It has also been suggested that PI cattle may suffer from platelet dysfunction; a presumed PI steer died of a fatal hemorrhage following castration [115]. Similarly, TI heifers display differential methylation of genes associated with bleeding and clotting time, although the genes altered in TI heifers are different from those altered in PI heifers. Alterations in the DNA of TI heifers at 4 months of age that indicate increased bleeding time include a hypermethylated CpG site within *CTBP2,* two within *EFNA5,* three within *GP6,* three within *KCNQ1,* three within *LIF,* two within *MUC2,* one within *PROZ,* eleven within *RXRA,* one within *THBS2,* four within *WNT7B,* one within *ALPL,* one within *CA5A,* one within *COL4A1,* one within *COL4A2,* and a single hypomethylated CpG site within *ATP12A* (Fig. 4A)*.* Differential methylation indicating altered clotting time in PI heifers was identified primarily in coagulation factors. No coagulation factors were differentially methylated in TI heifers. Further research will be necessary to conclude whether TI heifers suffer from disturbances to the coagulation cascade.

### Potential confounders and future research

As with all research, it is important to note potentially confounding variables related to the data. While peripheral WBCs are not the canonical choice to observe potentially pathological differences in DNA methylation, primary and secondary lymphoid tissue is unobtainable from a live animal. Peripheral WBCs were chosen for this study, not because they best represent the immune response, but because they represent a facet of the immune system that can be obtained without the need for euthanasia. By doing so, the monetary value of the experimental subject is preserved and the data produced is more suitable for application in production settings. Peripheral WBCs can be heterogeneous in cellular composition, which was verified by the finding of elevated intermediate monocytes and B cells by flow cytometry. As such, it is possible that a portion of the identified DMSs or DMRs were biased by increased concentrations of specific cell types. However, multiple steps were taken to assess and minimize the potential for bias within this study. Pearson correlation coefficients between all samples were greater than 0.94, coverage values between samples were normalized by the median, and the threshold for the identification of DMSs was set to 25% to mitigate potential sources of bias.

It is also important to note that the identification of increased methylation within a gene body, promoter, or CpG island is only associated with decreased expression, the impact is not implicit; the same is especially true for sites with decreased methylation, indicative of increased expression potential. Not all genes are always being actively transcribed. For example, hypermethylation within *MX1* may have no impact upon a calf that is not actively responding to an infection. If the same calf *were* to be infected with a pathogen, it is more possible that hypermethylation within *MX1* would decrease the calf’s ability to mount an effective immune response. Additional research is required to validate and confirm whether the differential methylation identified herein is truly translational to observed pathologies.

## Conclusion

**Fig. 6:**
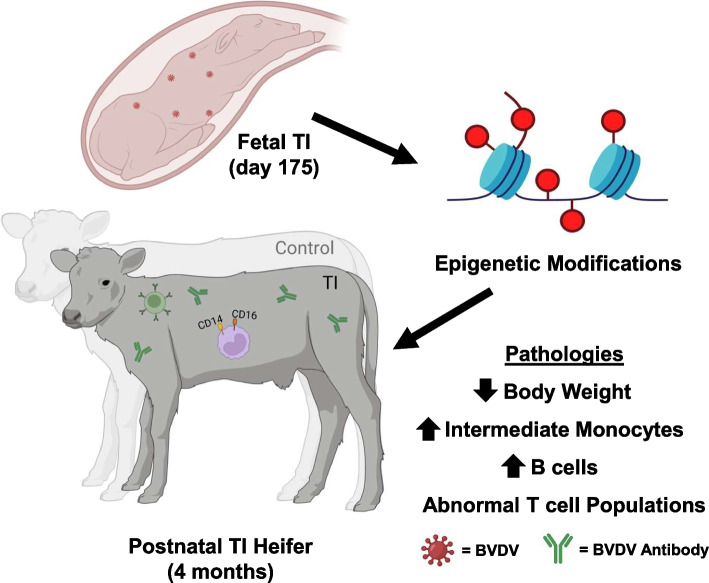
Transient fetal infection with BVDV results in epigenetic alterations and differences in intermediate monocytes, B cells, and body weight. A graphical summary of the findings identified in postnatal heifers following transient fetal infection with BVDV. The findings include decreased body weight as well as elevated intermediate monocytes and B cells

At 4 months of age, TI heifers exhibit increased circulating lymphocyte percentage, intermediate monocytes, B cells, CD4^+^/CD8b^+^ T cells, and CD25^+^/CD127^−^ T cells. Analysis of differential methylation in WBC DNA indicates abnormal regulation of genes involved in inflammation, ROS production, and insulin secretion (Fig. 6). Increased ROS production and inflammation are complementary responses to tissue injury and are related to the development of cancers, arthritis, as well as cardiac and metabolic disease. Further research concerning postnatal aberrations identified in TI cattle is crucial to the development of an accurate estimate of profit loss to BVDV which demonstrates the continued importance of the virus. A better understanding of how late gestation BVDV infections influence postnatal metabolism could allow for the application of therapeutics or management strategies to mitigate profit loss incurred by TI calves through decreased weight gain. Comparison of differential methylation identified in TI heifers to that found in PI heifers indicates some similarity in immunosuppression. The immunocompromised state of PIs is likely driven by constant infection, while immunosuppression in TI calves appears to be more related to chronic inflammation and over-responsiveness of facets of the immune system. Comparison of genes containing differentially methylated CpG sites indicates the persistence of methylation variation from birth to 4 months of age. If specific CpG sites are persistently differentially methylated over time, epigenetic marks may have the potential to serve as a biomarker for the identification of TI calves. If late gestation TI calves can be identified, the profit loss they induce can be managed through intervention strategies.

## Supplementary Information

Supplementary Material 1.

Supplementary Material 2.

Supplementary Material 3.

Supplementary Material 4.

Supplementary Material 5.

Supplementary Material 6.

Supplementary Material 7.

## Data Availability

The DNA methylome dataset analyzed within the current study is available within the NCBI GEO Database (GSE288374).

## Abbreviations

BVDV: Bovine viral diarrhea virus
PI: Persistently infected
TI: Transiently infected
WBC: White blood cell
RRBS: Reduced representation bisulfite sequencing
DMS: Differentially methylated site
ROS: Reactive oxygen species
CBC: Complete blood count
CpG: Cytosine-phosphate-guanine
NCP: Non-cytopathic
IPA: Ingenuity pathway analysis
ML: Machine learning
CI: Congenitally infected
