# Supplementary material for: Postnatal epigenetic differences in calves following transient fetal infection with bovine viral diarrhea virus
Source: BMC Genomics. 2025 May 2;26:441. doi: 10.1186/s12864-025-11562-5 (PMC12049026; doi:10.1186/s12864-025-11562-5)
Supplement: Supplementary file 1 — Supplementary Material 1. [file 12864_2025_11562_MOESM1_ESM.pdf]

# Ingenuity pathway analysis predicted canonical pathways

Downregulated  No change  Upregulated  No overlap with dataset

Green denotes hypermethylation while red denotes hypomethylation. The total number of genes included in the IPA pathway is denoted to the right of each stacked bar.

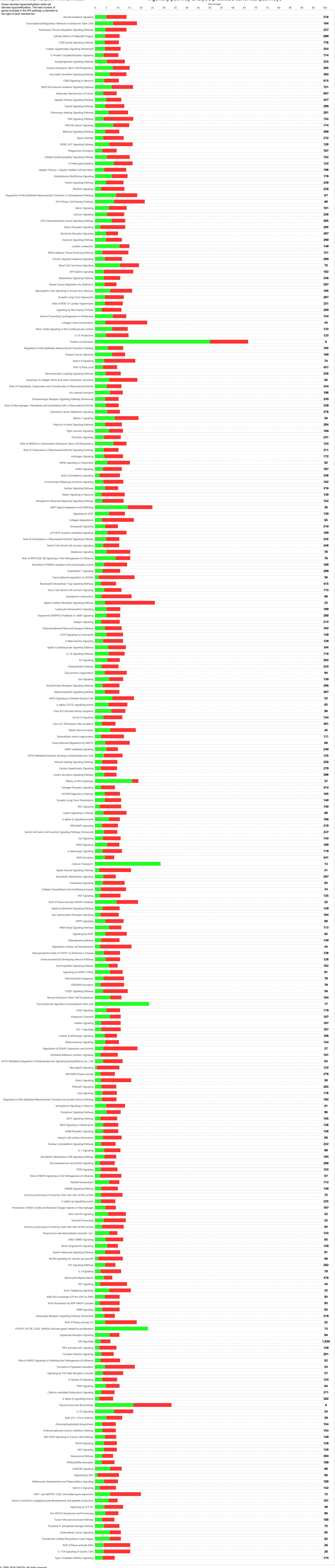


© 2000-2024 QIAGEN. All rights reserved.
