# Supplementary material for: Postnatal epigenetic differences in calves following transient fetal infection with bovine viral diarrhea virus
Source: BMC Genomics. 2025 May 2;26:441. doi: 10.1186/s12864-025-11562-5 (PMC12049026; doi:10.1186/s12864-025-11562-5)
Supplement: Supplementary file 2 — Supplementary Material 2. [file 12864_2025_11562_MOESM2_ESM.pdf]

Ingenuity pathway analysis machine learning disease pathways

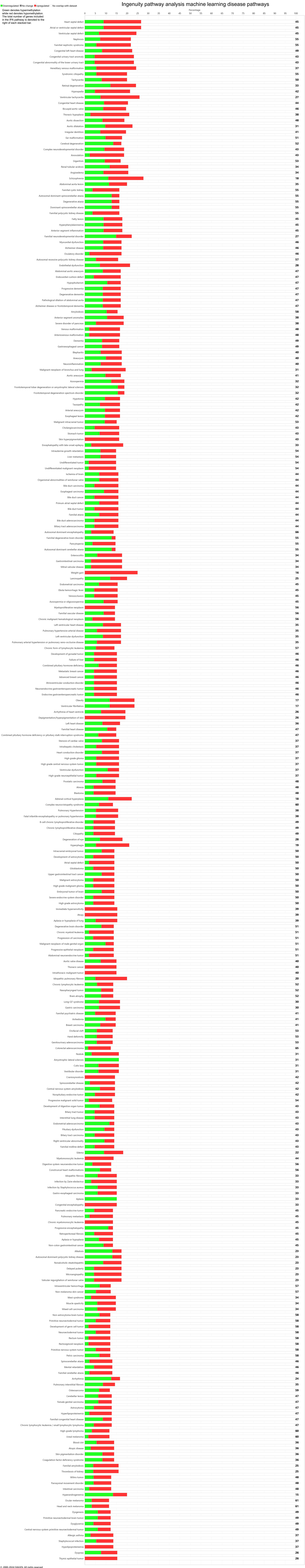

© 2000-2024 QIAGEN. All rights reserved.
